# Laryngology and Rhinology

**Published:** 1891-02

**Authors:** Frank Hamilton Potter


### Progress in Medical Science.

### LARYNGOLOGY AND RHINOLOGY.

CONDUCTED BY

FRANK HAMILTON, POTTER, M. D.,

### DIAGNOSIS AND THERAPEUTICS OF CANCER OF THE LARYNX.

Butlin, of London, (*Journal of Laryngology and Rhinology*, *Nov.*, 1890,) presented a paper on this subject before the Tenth International Medical Congress. He accepts the division proposed by Krishaber into extrinsic and intrinsic cancers. Extrinsic are those neoplasms which are situated on the arytenoid cartilages, ary-epiglottic folds, epiglottis and in the sinus pyriformis. Intrinsic are those situated on the true and false ligaments, ventricles and in the sub-glottic space. This division is of great practical value. Whilst the extrinsic forms have a rapid and bad progress, the intrinsic growths are limited for a longer period to the interior of the larynx, and have no great tendency to affect the lymphatic glands. The operation of total or partial extirpation of the larynx must only be practised in cases of intrinsic carcinoma, for only in such cases are successes observed. In one hundred and two collected cases of intrinsic cancer are twenty-eight cases of thyrotomy, twenty-three of partial, fifty-one of total extirpation, with three, seven, and sixteen deaths respectively from shock, septicemia, or *Schluck-pneumonie*. The author regards those cases as cured which last longer than three years without recurrence. The total results, then, so far as cured cases are concerned, are that fifteen patients were alive and free from disease, or died of some other disease than cancer, at periods of from three to twenty years after the last operation. Of the results of operations for recurrent disease, but little that is good can be said. The operations proved fatal in more than one of the cases, and not one of the patients received decided benefit. With regard to the choice of operation in individual cases, the smallest operation consistent with the widest excision of the disease, and the removal of a wide area of surrounding tissues, should be performed. Intralaryngeal operations, in spite of the good results obtained by B. Fränkel, can only be performed in few cases, because usually the cancer is more extensive than can be seen by the laryngoscope.

---

### ATROPHIC RHINITIS.

In our last report we called attention to some of the undecided questions in connection with this disease. We now present the conclusion of Seifert of Würzburg (*same*) as perhaps the latest contribution to the subject. In forty-nine cases of rhinitis atrophicans simplex the author observed only atrophy of the inferior turbinateds, whilst the middle turbinateds were hypertrophic. There was no fetor or formation of crusts. The same process existed in the pharynx as in the nose. In four cases the author examined pieces of the middle turbinateds, and found cylindrical or cuboidal epithelial-celled infiltration of the uppermost regions of the mucous membrane, and in the deeper parts hypertrophy of the fibrous tissue. In forty-five cases of the fetid form the lower turbinateds were also atrophic, and the middle turbinateds thickened. Tuberculosis or syphilis were rare. In slighter cases the epithelium was cylindrical, sometimes cuboidal, and in severe cases there was only plaster-epithelium, infiltrations of the glands or atrophy of these latter. The author believes in consequence of his microscopical examinations, that the slight and the severe forms are chronic catarrhs with degenerative alteratives of the mucous membranes. The fetor is the consequence of putrescence of the secretions.

---

### THE ANATOMY OF THE NASAL ERECTILE TISSUE.

J. Herzfeld, Berlin, (*Arch. J. Micros.*; Anat., Vol. XXXIV., p. 197,) in a contribution upon the anatomy of the erectile tissue of the nasal mucous membrane, draws the following conclusions in relation to it: *a.* The erectile tissue of the nasal mucous membrane is rich in organic muscle. This does not lie free in the areolar tissue of the erectile body, but, as in other situations, forms the tunica media of the arteries and veins and, further, lies closely packed about the lacunæ of the erectile body.

*b.* In the areolar tissue of the erectile body there are a number of elastic fibres.

*c.* The turbinate bone is spongy, containing marrow. The erectile tissue does not extend into the bone, but veins come from the bone, and anastomose with the veins of the periosteum and the outlet channels of the erectile tissue.—*Archives of Otology.*

---

### THE DEVELOPMENT OF THE NASAL CAVITY AFTER BIRTH.

Disse, J., of Berlin, (*Arch. f. Anat. u. Physiol.*, anat. Atth., Suppl. Band, p. 29, 1889,) has investigated this important question. The material studied comprised the skulls of twenty-six children, and twelve adults. The point chiefly considered in the investigations was the question why it is that difficulty in breathing—and, in consequence of this threatening symptoms from want of air—occurs so often under certain conditions in children, and so rarely in adults. The results were as follows: The structure of the nasal cavity in infants and children differs considerably from that in adults. The cavity is low in the new-born; it develops downward, and thus the olfactory region is removed from the floor of the cavity. The respiratory region increases in breadth and height. While the whole nasal cavity increases two and a half times in size, the respiratory region alone increases three times in its growth from birth up to the state of complete development. The increase in width during the same period is still more considerable.—*Archives of Otology.*

---

### THE RHEUMATIC AND GOUTY DIATHESIS AS MANIFESTED IN DISEASES OF THE THROAT.

Robinson, of New York, (*New York Medical Record*, Dec. 6, 1890,) in an article upon this subject, first describes the conditions of the throat that may be found in persons of the rheumatic diathesis and then reviews the treatment of these conditions. On the latter point he states that there is a rational basis for the treatment of these cases. In the first place the blood is subalkaline from retention of the excrementitious substances which should normally be expelled. In the second place the aliments taken as food are not properly oxidized and do not, therefore, take forms like urea, which are soluble and easily eliminated. Two objects, therefore, must be kept in view: I. The depuration of the blood. II. The increased oxidation of alimentary substances introduced into the stomach. The first object should be attained by the use of alkaline diuretics, by purgatives, by increasing eliminations through the skin, which can be accomplished by exercise, baths, friction. The second object must be attained by the use of iron, oxygen—breathing fresh, pure air. Among the alkaline diuretics the natural Vichy water is one of the best. Among the purgatives are recommended small doses of calomel, podophyllin, and Carlsbad Sprudel salts, dissolved in warm water or in the Sprudel water itself. Under certain conditions the Turkish baths, combined with exercise and friction, are of especial value.

Iron as the main carrier or oxygen to the economy should be given in small doses, frequently repeated and continued for a long time. Diet must be carefully regulated and particularly the excessive use of the albumenoid or starchy foods must be prevented. The internal use of the French sulphur waters, especially those of Aix-les-Bains in Savoy have unquestionably benefited some patients and are advised, but those of Sharon and Richfield in this country are to be avoided. They probably contain a large proportion of the insoluble salts of lime, and these, instead of favoring the elimination of the gouty products, cause their retention. Colchicum and its alkaloid colchicine are also advised in properly selected cases.

For the local treatment in the large majority of cases soothing sprays and inhalation are to be used and the astringent solutions avoided. Alkaline sprays with carbolic acid, thymol, menthol and warm inhalation with benzoin and fir-wood oil are often of great benefit. The latter should be given only at night, as, if given the day, they tend to increase the congestion and sensitiveness on account of the atmospheric changes to which the patient is of necessity exposed. Inhalation and pulverizations of sulphur water, together with pine needle extract, have also been found of great value.

---

### A NEW METHOD OF RAISING THE EPIGLOTTIS.

PRESTON (*Allgemeine Wiener mediz. Zeitung*, No. 13, 1890,) describes a method of raising an overhanging epiglottis, which, from its simplicity, will at once commend itself. He takes a laryngeal probe and bends its lower extremity laterally to the extent of one and a half centimetres. This is then passed along the dorsum of the tongue in such a manner as to depress the median glossoepiglottic fold. This will raise the epiglottis so as not only to expose the vocal cords in their entire length, but also the lower surface of the epiglottis itself. This manipulation causes but little irritation, and can be endured during an ordinary laryngeal operation.

